# Evaluation of PEEP and prone positioning in early COVID-19 ARDS

**DOI:** 10.1016/j.eclinm.2020.100579

**Published:** 2020-10-11

**Authors:** Mirja Mittermaier, Philipp Pickerodt, Florian Kurth, Laure Bosquillon de Jarcy, Alexander Uhrig, Carmen Garcia, Felix Machleidt, Panagiotis Pergantis, Susanne Weber, Yaosi Li, Astrid Breitbart, Felix Bremer, Philipp Knape, Marc Dewey, Felix Doellinger, Steffen Weber-Carstens, Arthur S. Slutsky, Wolfgang M. Kuebler, Norbert Suttorp, Holger Müller-Redetzky

**Affiliations:** a Department of Infectious Diseases and Respiratory Medicine, Charité – Universitätsmedizin Berlin, corporate member of Freie Universität Berlin, Humboldt-Universität zu Berlin, and Berlin Institute of Health, Berlin, Germany; b Berlin Institute of Health, Berlin, Germany; c Department of Anesthesiology and Operative Intensive Care Medicine (CCM, CVK), Charité – Universitätsmedizin Berlin, corporate member of Freie Universität Berlin, Humboldt-Universität zu Berlin, and Berlin Institute of Health, Berlin, Germany; d Department of Radiology, Charité – Universitätsmedizin Berlin, corporate member of Freie Universität Berlin, Humboldt-Universität zu Berlin, and Berlin Institute of Health, Berlin, Germany; e Interdepartmental Division of Critical Care Medicine, University of Toronto, Toronto, ON, Canada; f Keenan Research Center for Biomedical Science, St. Michael's Hospital, Toronto, Ontario, Canada; g Charité – Universitätsmedizin Berlin, corporate member of Freie Universität Berlin, Humboldt-Universität zu Berlin, and Berlin Institute of Health, Institute of Physiology, Berlin, Germany; h Department of Surgery and Physiology, University of Toronto, Toronto, Ontario, Canada; i Division of Pulmonary Inflammation, Charité – Universitätsmedizin Berlin, corporate member of Freie Universität Berlin, Humboldt-Universität zu Berlin, and Berlin Institute of Health, Berlin, Germany

## Abstract

**Background:**

In face of the Coronavirus Disease (COVID)-19 pandemic, best practice for mechanical ventilation in COVID-19 associated Acute Respiratory Distress Syndrome (ARDS) is intensely debated. Specifically, the rationale for high positive end-expiratory pressure (PEEP) and prone positioning in early COVID-19 ARDS has been questioned.

**Methods:**

The first 23 consecutive patients with COVID-19 associated respiratory failure transferred to a single ICU were assessed. Eight were excluded: five were not invasively ventilated and three received veno-venous ECMO support. The remaining 15 were assessed over the first 15 days of mechanical ventilation. Best PEEP was defined by maximal oxygenation and was determined by structured decremental PEEP trials comprising the monitoring of oxygenation, airway pressures and trans-pulmonary pressures. In nine patients the impact of prone positioning on oxygenation was investigated. Additionally, the effects of high PEEP and prone positioning on pulmonary opacities in serial chest x-rays were determined by applying a semiquantitative scoring-system. This investigation is part of the prospective observational PA-COVID-19 study.

**Findings:**

Patients responded to initiation of invasive high PEEP ventilation with markedly improved oxygenation, which was accompanied by reduced pulmonary opacities within 6 h of mechanical ventilation. Decremental PEEP trials confirmed the need for high PEEP (17.9 (SD ± 3.9) mbar) for optimal oxygenation, while driving pressures remained low. Prone positioning substantially increased oxygenation (*p*<0.01).

**Interpretation:**

In early COVID-19 ARDS, substantial PEEP values were required for optimizing oxygenation. Pulmonary opacities resolved during mechanical ventilation with high PEEP suggesting recruitment of lung volume.

**Funding:**

German Research Foundation, German Federal Ministry of Education and Research.

**Research in context**

**Evidence before this study**

We searched PubMed without language restriction for studies published from database inception until August 15th, 2020, with the terms “SARS-CoV-2″ or ”COVID-19″ and “ARDS” or “mechanical ventilation” or “PEEP” or “prone positioning” or “respiratory failure” and found no relevant articles pertaining to COVID-19 ARDS. To our knowledge, there have been no reports on PEEP titration in conjunction with the assessment of radiographic changes and lung mechanics in early COVID-19 ARDS.

**Added value of this study**

We demonstrate that patients with early COVID-19 ARDS can benefit in terms of oxygenation from mechanical ventilation with high PEEP as well as from prone positioning.

**Implications of all the available evidence**

COVID-19 ARDS lung exhibits a remarkable high lung compliance but despite its unique nature we show here that COVID-19 ARDS patients benefit from high PEEP and respond well to prone positioning regarding oxygenation. Our findings provide evidence that may help guide intensivists in the treatment of early COVID-19 ARDS.

Alt-text: Unlabelled box

## 1 Introduction

Coronavirus disease 2019 (COVID-19), caused by infection with the severe acute respiratory syndrome coronavirus 2 (SARS-CoV-2), is a global health emergency. The progression to respiratory failure and the requirement for mechanical ventilation in some patients has pushed health care systems worldwide to or beyond their limits [1]. The burden of disease is still on a rising trajectory in most parts of the world [2].

Approximately 17 percent of patients with COVID-19 pneumonia require invasive mechanical ventilation [3,4]. While the COVID-19 phenotype has recently been proposed to be similar to high-altitude pulmonary edema [5] or to represent a novel class, [6] COVID-19 induced pneumonia often fulfills all criteria of the acute respiratory distress syndrome (ARDS) as defined by the Berlin definition [7]. In line with the classic pathology of ARDS, [8] these clinical findings are associated with a hyperinflammatory response in the lungs of COVID-19 patients evident as distinct inflammatory cell infiltration and diffuse alveolar damage [9].

A series of editorials and case reports recently proposed that principles of mechanical ventilation in early COVID-19 should deviate from classic ARDS [10], [11], [12], [13]. This suggestion is based on the notion that some COVID-19 ARDS patients (“L-type”) are characterized by low lung elastance, low lung weight, and low lung recruitability; and the ”H-type” are characterized by high lung elastance, high intrapulmonary right-to-left shunt, high lung weight, and high lung recruitability, typical for classic ARDS. Based on the l-type characteristics, the editorialists have argued for more liberal tidal volumes and against the use of high positive end-expiratory pressure (PEEP) in early COVID-19 ARDS. As well, the usefulness of prone positioning in early COVID-19 ARDS has been questioned. These editorials have generated considerable debate regarding optimal ventilatory strategies for COVID-19 ARDS.

In this prospective study we set out to address this debate by examining the effects of PEEP titration and prone positioning in the first cohort of early COVID-19 ARDS patients transferred to our ICU. We studied detailed physiological responses to PEEP maneuvers and prone position in these patients. Our findings suggest that patients with early COVID-19 ARDS do not differ in their response to high PEEP and prone positioning from classic ARDS, and should therefore be ventilated according to established ARDS principles and regimens.

## 2 Methods

### 2.1 Study design and oversight

Between March 15 and April 11, 2020 we enrolled the initial cohort of 23 consecutive patients with COVID-19 ARDS admitted to one of the three ICUs of the Charité - Universitätsmedizin Berlin ARDS/ECMO Center as part of the prospective, observational PA-COVID-19 trial on the pathophysiology and clinical course of COVID-19 (retrospectively registered in the German clinical trials register on May 13, 2020 (ID: DRKS00021688) [14]. The study was approved by the ethics committee of the Charité - Universitätsmedizin Berlin (EA2/066/20) and was performed according to the Declaration of Helsinki and Good Clinical Practice principles (ICH 1996). Written informed consent was obtained from all patients, or their legal representatives.

### 2.2 Study population

**Fig. 1:**
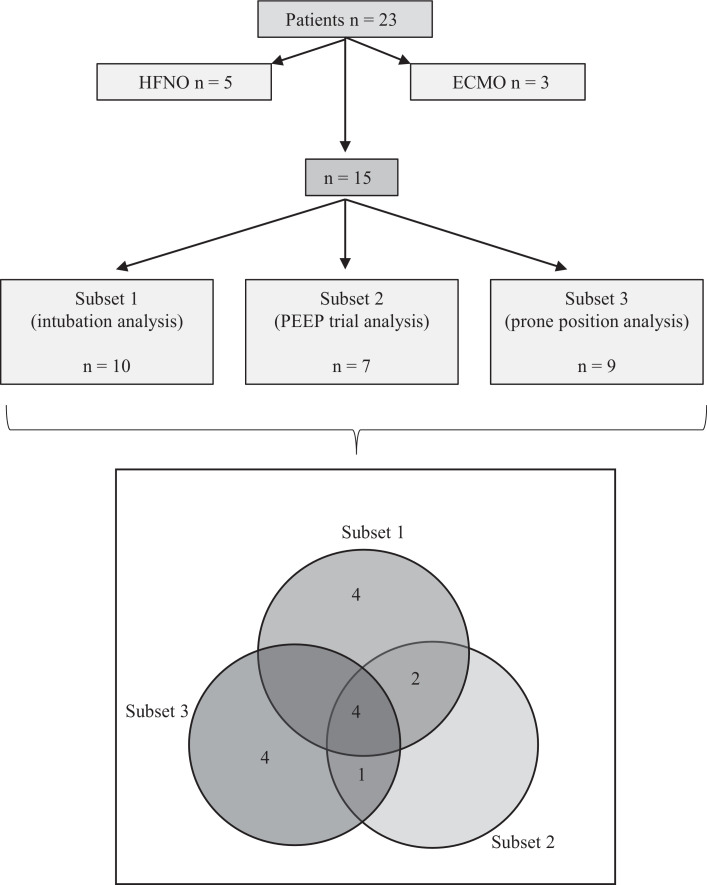
**Study cohort flowchart. A,** The first consecutive 23 COVID-19 patients treated on our ICU were enrolled to this study, investigating PEEP and prone positioning in mechanically ventilated patients. Eight patients were excluded from the assessment of the specific interventions as they received ECMO therapy (three patients, ECMO therapy would interfere with the analysis of oxygenation), or high-flow oxygen therapy but no mechanical ventilation (five patients that could not be asses regarding the effects of invasive mechanical ventilation, PEEP and prone positioning), resulting in 15 patients eligible for this study. Ten patients were intubated in our ICU (Subset 1, intubation group), while eight patients were transferred intubated to our ward. For seven of the 15 mechanically ventilated patients, a full dataset of decremental PEEP trials was available and they were accordingly included in Subset 2 (PEEP trial analysis) for determination of optimal PEEP. Nine of the fifteen patients were subject to prone positioning (Subset 3, prone position analysis). **B,** Illustration of the allocation of the 15 patients to each analysis.

All 23 patients were referred to the ICU of a tertiary referral center with respiratory failure following positive polymerase chain reaction (PCR) SARS-CoV-2 infection. All 23 patients were included in the descriptive analysis of the cohort. For the analysis of the effect of mechanical ventilation with high PEEP on recruitment and oxygenation, PEEP titration and effect of prone positioning the three patients on veno-venous extracorporeal membrane oxygenation (vvECMO) were excluded as ECMO therapy was judged to be a significant bias in the analysis of oxygenation. Five patients were excluded as they were treated with high flow nasal oxygen, and did not receive mechanical ventilation. The remaining 15 mechanically ventilated patients were studied for up to 15 days following intubation. All ten patients intubated in our ICU were included in Subset 1 (intubation group) to assess the initial effects of invasive mechanical ventilation on oxygenation (four patients were intubated due to severe oxygenation failure, 6 were intubated due to severe oxygenation failure and concomitant high ventilatory effort). For seven of the 15 mechanically ventilated patients a full dataset of decremental PEEP trials was available and they were accordingly included in Subset 2 (PEEP trial group) to determine best PEEP. Nine of the 15 patients were place prone within the first 3.0 (SD ± 3.9) days following intubation and were included in Subset 3 (prone position group) to assess the effect of prone positioning. Fig. 1 details the assignment of all 23 patients. Of all patients, 15 patients were transferred from the emergency room or regular wards within the hospital of which ten were intubated in our ICU. Eight patients were transferred intubated to our ICU from regional secondary hospitals including the three vvECMO patients. Demographic and clinical characteristics of the patients at admission are listed in Table 1.

**Table 1:** Demographic and clinical characteristics of the patients.

Demographic and clinical characteristics of the patients	
Characteristics	
Patients	*n* = 23
Age (years)	62·1 ± 14·1
Age (range)	26 - 81
Sex - Male (%)	73·9
BMI, kg/m^2^	29·3 ± 4·8
Mean duration of symptoms before admission (days)	6·4 ± 3·2
Mean duration of symptoms before intubation (days)	7·6 ± 3·7
Number of patients with high-flow nasal oxygen (no intubation)	5
Number of patients with ECMO	3
Hospital mortality rate (%) *	29·2
Hospital mortality rate of patients with high-flow nasal oxygen (%)	20·0
Hospital mortality rate of ECMO patients (%)	0·0
Length of Stay (days)*	35·7 ± 32·2
Length of Stay of patients with high-flow nasal oxygen (days)	14·2 ± 5·8
Length of Stay of ECMO patients (days)	70·7 ± 36·9
Percentage of all patients who received intermittently muscle relaxants (%)	30·4
Percentage of ECMO patients who received intermittently muscle relaxants (%)	100·0
Percentage of all patients who received intermittently norepinephrine and vasopressin (%)	30·4
Percentage of ECMO patients who received intermittently norepinephrine and vasopressin (%)	66·7
APACHE II	18·0 ± 9·4
SOFA all patients	6·3 ± 4·9
SOFA of patients with high-flow nasal oxygen (no intubation)	2·2 ± 1·3
SOFA of patients with ECMO	11·0 ± 5·3
COVID-19 symptoms - no. (%)	
Dyspnea	19 (82·6)
Cough	18 (78·3)
Sore throat	3 (13)
Fever	22 (95·7)
Myalgia	9 (39·1)
Exhaustion	13 (56·5)
Coexisting conditions - no. (%)	
COPD	3 (13·0)
Asthma	1 (4·3)
Other lung disease	3 (13·0)
Pre-obesity	10 (43·5)
Obesity	8 (34·8)
Arterial hypertension	18 (78·3)
Diabetes mellitus Type 2	9 (39·1)
Coronary heart disease	2 (8·7)
HIV, transplantation or immuno-supressive medications	2 (8·7)
**Subset 1 (Intubation group)**	
Patients	10
Age (years)	70·4 ± 10·8
Sex - Male (%)	80
BMI, kg/m^2^	28·4 ± 4·5
Mean duration of symptoms before admission (days)	7·2 ± 4·3
Mean duration of symptoms before intubation (days)	8·7 ± 4·2
Hospital mortality rate (%)	40·0
Length of Stay (days)*	38·3 ± 26·1
Percentage of patients who received intermittently muscle relaxants (%)	40·0
Percentage of patients who received intermittently norepinephrine and vasopressin (%)	30·0
APACHE II	17·8 ± 9·4
SOFA	3·9 ± 2·9
**Subset 2 (PEEP trial group)**	
Patients	7
Age (years)	61·4 ± 15·8
Sex - Male (%)	57·1
BMI, kg/m^2^	30·0 ± 6·5
Mean duration of symptoms before admission (days)	5·7 ± 2·6
Mean duration of symptoms before intubation (days)	6·7 ± 2·7
Hospital mortality rate (%)	42·9
Length of Stay (days)	56·3 ± 32·9
Percentage of patients who received intermittently muscle relaxants (%)	28·6
Percentage of patients who received intermittently norepinephrine and vasopressin (%)	28·6
APACHE II	20·7 ± 8·8
SOFA	5·7 ± 5·2
Respiratory rate/ min	16·0 ± 2·6
**Subset 3 (Prone position group)**	
Patients	9
Age (years)	62·0 ± 14·2
Sex - Male (%)	66
BMI, kg/m^2^	30·4 ± 6·5
Mean duration of symptoms before admissions (days)	6·7 ± 4·8
Mean duration of symptoms before intubation (days)	7·2 ± 4·8
Hospital mortality rate (%)	55·6
Length of Stay (days)^a^	50·4 ± 34·9
Percentage of patients who received intermittently muscle relaxants (%)	55·6
Percentage of patients who received intermittently norepinephrine and vasopressin (%)	44·4
APACHE II	26·2 ± 6·5
SOFA	7·4 ± 4·9
Mean time to first prone positioning (days)	3 ± 3·9
Duration of each prone positioning (hours)	15·4 ± 2·5

Values are means ± SD. COPD, chronic obstructive pulmonary disease; COVID-19, coronavirus disease 2019; APACHE II, Acute Physiology And Chronic Health Evaluation II; SOFA, Sepsis-related organ failure assessment score. APACHE II and SOFA score were obtained at admissions day. #One patient is still hospitalized and thus excluded from the hospital mortality rate.

a One patient is still hospitalized and excluded from hospital mortality rate and length of stay analyses.

### 2.3 Mechanical ventilation and decremental PEEP trials

The initial PEEP after intubation was determined using the ARDSnet high PEEP table [15]. In patients receiving low flow oxygen prior to intubation, the F_i_O_2_ necessary to maintain adequate oxygenation was calculated from the demand of supplemental oxygen [16]. In patients treated with high flow nasal oxygen (HFNO), the adjusted F_i_O2 was used. Notably high minute ventilation under supplemental oxygen even when delivered as HFNO may lower the effective F_i_O_2_. Further hyperventilation results in decreased alveolar CO_2_ and vice versa increased alveolar O_2_ which would improve oxygenation by raising the diffusion gradient for oxygen. In the current setting the estimated F_i_O_2_ could not be corrected for both opposing interrelations. In general, PEEP values 2 mbar higher than the value suggested by the higher PEEP / lower F_i_O_2_ table of the ARDS Network were initially applied after intubation (Supplementary Table S1) [15]. In patient 2, the initial PEEP was set lower as blood oxygenation increased quickly at this PEEP level after intubation. Patients were ventilated in either volume or pressure controlled mode with a tidal volume targeting 6 ml/kg predicted body weight. Respiratory rate was set to facilitate CO_2_ removal while preventing dynamic hyperinflation, and permissive hypercapnia was tolerated if necessary to adhere to lung protective ventilation. Assisted spontaneous breathing was allowed after PEEP titration, when considered safe by the attending physician. An esophageal catheter (, NutriVent™,Sidam Group, San Glacomo Roncole, Italy) was placed allowing to measure the esophageal pressure by connecting the balloon filled with 4 ml of air to the corresponding pressure port of the ventilator (Hamilton S1, Hamilton Medical, Bonaduz, Switzerland). Position of the balloon in the mid to lower third of the thorax was validated by typical cardiac oscillations, dynamic occlusion tests and by chest x-ray (CXR). Transpulmonary pressure at end-inspiration and end-expiration were calculated directly from simultaneously measured airway and esophagus pressures.

PEEP trials were performed applying volume controlled ventilation with constant respiratory rate, F_i_O_2_ and tidal volume (V_T_; 6.4 (SD ± 0.5) ml/kg predicted bodyweight). After a stabilization period of at least 10 min, PEEP was reduced stepwise in 2 mbar decrements. Prior to each PEEP reduction, arterial blood gas analysis was performed and respiratory parameters (as detailed in Table 2 and Supplementary Table S2) were recorded including P_a_O2, P_a_CO2, end-inspiratory airway plateau airway pressure, end-inspiratory esophageal pressure, end-expiratory esophageal pressure and respiratory system compliance (C_RS_). Driving pressure (ΔP= end inspiratory airway plateau pressure - PEEP), inspiratory trans-pulmonary pressure (T_Pi_= end-inspiratory airway plateau pressure – end-inspiratory esophagus pressure), end-expiratory trans-pulmonary pressure (T_Pexp_= PEEP - end-expiratory esophageal pressure), trans-pulmonary driving pressure (ΔT_P_= (T_Pi_-T_Pexp_)), were calculated. Optimal PEEP was defined at the PEEP corresponding to the maximal P_a_O_2_/F_i_O_2_. Downward PEEP titration was stopped when P_a_O_2_ decreased. In general, under mechanical ventilation target oxygen saturation was 90–95%, in patients receiving vasopressor support a mean arterial pressure of 65–70 mmHg was adjusted. Lactate levels were measured at least 3 times a day and transthoracic echocardiography was regularly performed to monitor cardiac function particularly when high airway pressures were applied.

**Table 2:** PEEP trials in early COVID-19 ARDS patients.

Patient:	1	2	3	4	5	6	7	Mean	SD	Median	Inter-quartile range
**F_i_O_2_ prior-intubation**	1	0·7	0·8	0·7	0·75	0·7	0·8	***0·8***	***0·1***	***0·75***	***0·1***
**Initial PEEP at start of PEEP trial (mbar)**	24	13	24	18	20	18	24	***20·1***	***3·9***	***20·0***	***6·0***
**Results PEEP Trials:**											
**Optimal PEEP (mbar)**	22.0	11.0	20.0	18.0	16.0	16.0	22.0	***17·9***	***3·6***	***18·0***	***5·0***
**V_T_ (ml/ kg)**	6·3	6·1	7·0	6·7	5·5	7·2	6·4	***6·4***	***0·5***	***6·4***	***0·53***
**Respiratory rate (min^−1^)**	13.0	15.0	13.0	15.0	22.0	16.0	16.0	***15·7***	***2·8***	***15·0***	***2·0***
**Respiratory system compliance (C_RS_) (ml/cmH_2_O)**	59·6	45·0	79.0	64·8	30.0	41·9	60·1	***54·3***	***15·2***	***59·6***	***19·0***
**Driving pressure (ΔP) (mbar)**	8.0	9.0	7.0	11.0	17·2	11.0	9.0	***10·3***	***3·1***	***9·0***	***2·5***
**Trans-pulmonary driving pressure (ΔTP) (mbar)**	6·9	7·9	4·5	7·1	11·8	8·4	6·1	***7·5***	***2·1***	***7·1***	***1·65***
**End-expiratory trans-pulmon-ary pressure (TP_exp_) (mbar)**	3·1	0·3	0·8	10.0	10·8	2·6	7·9	***5·1***	***4·1***	***3·1***	***7·25***
**F_i_O_2_**	0·5	0·4	0·3	0·35	0·5	0·6	0·35	***0·4***	***0·1***	***0·4***	***0·15***
**P_a_O_2_ (mmHg)**	85.0	85·0	71·0	65·3	81·7	79·6	88·7	***79·5***	***7·8***	***81·7***	***9·7***
**P_a_CO_2_ (mmHg)**	59.0	44·0	50·0	47·3	73·4	45·0	48·4	***52·4***	***9·7***	***48·4***	***8·35***
**P_a_O_2_/F_i_O_2_ ratio (mmHg)**	170·0	242·9	236·7	186·6	163·4	132·7	253·4	***197·9***	***43·0***	***186·6***	***78·35***

Results of PEEP trial shows the optimal PEEP as determined by decremental PEEP trial, and corresponding respiratory system mechanics and arterial blood gas analyses for all seven patients, as well as means, standard deviations (SD), median, and interquartile range.

### 2.4 Prone maneuver

Prone positioning was applied when P_a_O_2_/F_i_O_2_ ratio was <150 mmHg (*n* = 10). Patients remained prone on average 15.4 (SD ± 2.5) h for 6.2 (SD ± 3.4) consecutive days. The P_a_O_2_/F_i_O_2_ ratio, oxygenation-index (OI; OI = F_i_O_2_ * mean airway pressure/P_a_O_2_), and PEEP (with optimal PEEP levels set by decremental PEEP trials in supine and prone position, respectively) were assessed after 4 h in the supine position (SP) or 12 h prone. Mean values ± standard deviation (SD) were compared after the first prone positioning (first supine positioning vs. first prone positioning), after the first three prone maneuvers (first three supine positioning vs. first three prone positioning), and of all prone positions received (all supine positioning vs. all prone positioning).

### 2.5 Radiologic studies

CXR taken in supine position 5.9 h (SD± 1.8) after the respective prone positioning were evaluated independently by two board-certified radiologists. To assess changes in pulmonary opacification after intubation and prone positioning, respectively, a CXR scoring system was applied as described previously [17].

### 2.6 Statistics

To analyze the CXR scoring system results, a Wilcoxon signed-rank test was used to compare the changes in mean opacity. Otherwise analyses were performed using a Student`s paired, two-tailed, *t*-test. A *p* value <0.05 was considered to indicate statistical significance. The normal distribution of the data was verified by histogram visualization. As an additional sensitivity analysis we applied a non-parametric test, thereby relaxing the assumption of normality of the parameter and found no relevant changes in the results. Statistical analyses were performed with GraphPad 8 Prism.

### 2.7 Role of the founding source

The funders had no role in study design, data collection, analysis, or interpretation of the data, or in the writing of this study. The corresponding author had full access to all of the data and the final responsibility to submit for publication.

## 3 Results

### 3.1 Invasive positive pressure ventilation improves oxygenation and reduces CXR opacities in early COVID-19 ARDS

**Fig. 2:**
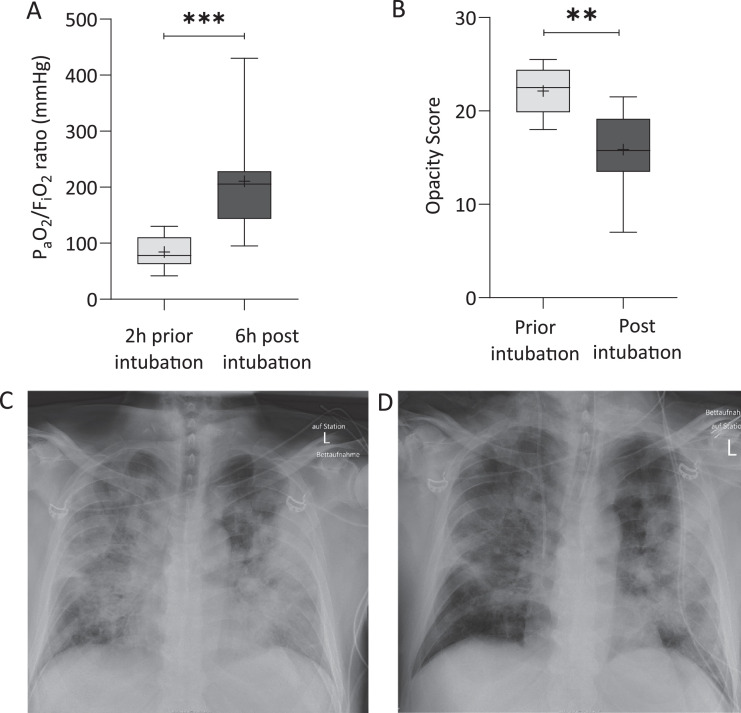
**Invasive positive pressure ventilation with high PEEP improves oxygenation and reduces opacities in chest x-rays.** The left boxplots (**A**) present the P_a_O_2_/F_i_O_2_ ratio two hours prior and six hours post intubation from the ten patients of Subset 1. Scoring of pulmonary opacities was performed, showing a reduction in pulmonary opacity scores with positive pressure ventilation (**B**). Representative chest x-ray images of one patient obtained before intubation (**C**) and after onset of mechanical ventilation (**D**) are shown. Whiskers indicate the 5th and 95th percentile. The cross within the box marks the mean. A two-sided paired *t*-test (A), and a Wilcoxon signed-rank test (B) was performed. **p<0·01, ***p<0·001.

In Subset 1 (intubation group), the 10 consecutive patients (age: 70·4 (SD ± 10·8) years; 80% male) were referred to our ICU after being symptomatic for 7·2 (SD ± 2·3) days. Following an initial, unsuccessful treatment with HFNO, patients were intubated 8·7 (SD ± 4·2) days after onset of symptoms (Table 1). Respiratory rate and P_a_CO_2_ prior to intubation were 31 (SD ± 2·6) per min and 35·9 (SD ± 7·0) mmHg; F_i_O_2_ required to maintain peripheral arterial oxygen saturation (SaO_2_) between 88 and 92% was 0·8 (SD ± 0·12) (Supplementary Table S1). After intubation and initiation of invasive positive pressure ventilation the P_a_O_2_/F_i_O_2_ ratio increased from 84·3 (SD ± 28) mmHg to 210·7 (SD ± 86·6) mmHg within 6 h of mechanical ventilation (Fig. 2), and the F_i_O_2_ required for a SaO_2_ between 88% and 92% decreased to 0·4 (SD ± 0·11) (for detailed respiratory parameters please see Table S2). CXR images obtained before intubation and 3 to 16 h after invasive ventilation revealed a reduction in opacities (*p* = 0·002, Fig. 2). Paired CXR images prior and post intubation are provided for each patient in online Supplementary Figure S1.

### 3.2 High PEEP is required for optimal oxygenation in early COVID-19 ARDS

In Subset 2 (PEEP trial group), decremental PEEP trials were performed in 7 ventilated patients to identify the optimal PEEP, defined as maximal P_a_O_2_/F_i_O_2_. PEEP trials were performed between 2 h and 60 h after initiation of mechanical ventilation. The optimal PEEP level was 17·9 (SD ± 3·6) mbar (Table 2), which was slightly above the corresponding recommended PEEP according to the ARDSnet high PEEP table (16 mbar for FiO_2_ of 0·4 – 0·5). Respiratory parameters and arterial blood gases for each step of the decremental PEEP trial and each individual patient are given in Supplementary Table S2.

### 3.3 Prone positioning improves oxygenation in early COVID-19 ARDS

**Fig. 3:**
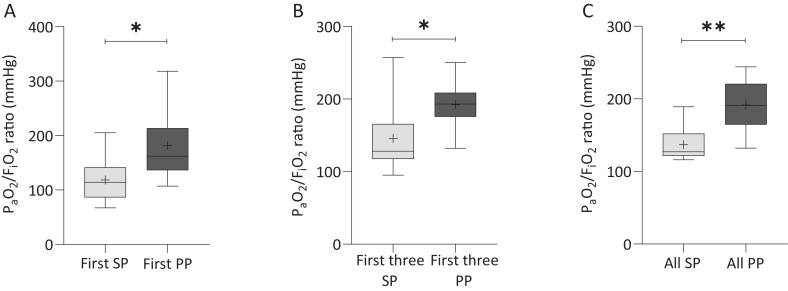
**Prone positioning improves oxygenation.** P_a_O_2_/F_i_O_2_ ratio was measured in nine patients subjected to prone positioning (Subset 3). Group data show P_a_O_2_/F_i_O_2_ ratio after the first prone positioning (PP) relative to the previous supine position (SP) (**A**), after the first three PP maneuvers vs. the three preceding SPs (**B**), and for all prone positions (**C**). Whiskers indicate the 5th and 95th percentile. The cross within the box marks the mean. A two-sided paired *t*-test was performed. *p<0·05, **p<0·01.

In Subset 3 (prone position group), 9 consecutive mechanically ventilated patients (62.0 (SD ± 14·2) years old, 66% male) were subjected to prone maneuvers. Patients had been symptomatic for 6·7 ± 4·8 days, were intubated 7·2 (SD ± 4·8) days after symptom onset, and had been ventilated for 3·0 (SD ± 3·9) days prior to first prone positioning (Table 1). In all analyses, P_a_O_2_/F_i_O_2_ ratio and OI were markedly improved in prone compared to the preceding supine position (*p*<0·01), while the optimal PEEP was slightly lower in prone positioning (*p*<0·05, Fig. 3, Supplementary Table S3). CXRs were obtained before and after the first prone maneuver in 7/9 patients, and before and after the second and third prone maneuver in 8/9 patients. CXR did not reveal a significant reduction in opacity score in response to prone positioning(after first prone positioning: *p* = 0·35, after third prone positioning: *p* = 0·37, Supplementary Fig. S2), suggesting that improved oxygenation did not primarily result from lung recruitment. CXR images of all patients before and after the first prone maneuver are provided in Supplementary Figure S2.

## 4 Discussion

In patients with early COVID-19 ARDS treated in our ICU, oxygenation improved markedly while radiographic pulmonary opacities decreased, after initiation of invasive mechanical ventilation. Decremental PEEP trials identified that high PEEP values of 18 (SD ± 4) mbar were required for optimal oxygenation. Individual optimal PEEP values were comparable to values suggested by the high ARDS network PEEP table [15]. Oxygenation increased reproducibly in response to repeated prone positioning as compared to supine positioning. Our findings suggest that patients with early COVID-19 ARDS do not differ in their response to high PEEP and prone positioning from classic ARDS, and should therefore be ventilated according to established ARDS principles and regimens.

Patients were referred to our ICU within 6.4 days (SD ± 3.2), and were intubated within 7.6 days (SD ± 3.7) after onset of symptoms. Regarding demographic characteristics and preexisting conditions the cohort was comparable to 1727 COVID-19 patients treated with mechanical ventilation on ICUs in Germany between 26. February 2020 and 19. April 2020 [18]. We did not use non-invasive ventilation, and patients were intubated if high flow nasal oxygen therapy was insufficient to ensure adequate oxygenation or if the patient remained tachypneic (respiratory rate >30 min^−1^) or hypocapnic despite being sufficiently oxygenated. Based on these characteristics, patients in this study can be considered to reflect cases of early COVID-19 ARDS.

A number of recent inter-related editorials have suggested that a subset of patients with COVID-19 induced ARDS have an unusual physiological phenotype (“L-type” phenotype), with low elastance, low lung weight, and low recruitability. Based on these physiological results the authors suggested that high levels of PEEP may be detrimental and that prone positioning is likely not indicated [11]. There is some question as to whether the respiratory mechanics of COVID-19 induced ARDS are indeed as heterogeneous or unusual as suggested in these editorials [10], [11], [12], [13]. Our case series does not directly address this particular question, as the number of patents we studied is quite small. However, we think this study is important because it directly addresses the conclusions of the editorialists with respect to what is the optimal ventilatory strategy for these patients.

The respiratory system mechanics of our patients are very close to those of Gattinoni et al. [10] with C_rs_ of 54.3 (SD ± 15.2) ml/cmH_2_O at optimal PEEP, compared to 50.2 (SD ± 14.3) ml/cmH_2_O in their study. As such we were studying a group of patients with very similar mechanical characteristics to what they described. We carefully titrated PEEP in our patients to avoid potential adverse effects of high PEEP levels in lungs with rather low elastance. That notwithstanding, we identified that high PEEP levels were required for optimized oxygenation, while driving pressure remained within margins commonly considered safe. We did not detect PEEP related hemodynamic instabilities (i.e. no increase of norepinephrine demand or lactate levels). Our results are not in accord with the recommendation of Gattinoni et al. who suggested relatively low PEEP levels of 8–10 cm H_2_O [19]. In addition, they recommended that the prone positioning should only be used as a rescue maneuver as the lungs of these patients were “too good” for the prone positioning to be effective; we found that these patients responded to the prone with improved oxygenation. It is important to point out their recommendations were based on a theoretical assessment informed by baseline lung mechanics, and not on detailed measurements of the consequences of higher PEEP or prone positioning in these patients.

The fact that high PEEP may markedly improve oxygenation in lungs with a low elastance may seem puzzling. Despite their low elastance, patients commonly presented with a high proportion of non-aerated lung tissue as recently reported by Lieuwe and coworkers in 70 patients [20]. This finding is in line with improved oxygenation in our patients upon initiation of mechanical ventilation with high PEEP, in association with partial resolution of wide-spread pulmonary opacities. In our study optimizing oxygenation was the main goal to be achieved by PEEP titration. Setting PEEP to optimize oxygenation is rational as optimal oxygenation results from an optimized gas exchange area stabilized by high PEEP that implies an increased functional residual capacity which would result in minimized end-inspiratory lung strain, minimized cyclic opening and closing of alveoli - atelectrauma, and an ideally homogenously ventilated lung which altogether should be protective against ventilator-induced lung injury [21,22,23]. However, aiming solely on optimized oxygenation by setting the PEEP contains the risk of PEEP levels too high resulting in potentially injurious lung stress, which would promote ventilator-induced lung injury and hemodynamic deterioration. This may be avoided by rigorously limiting driving pressures and conscientious hemodynamic monitoring which we did during PEEP titration.

The observed marked improvement in oxygenation and recruitment of lung volume with high PEEP is in line with the study of van der Zee in which electric impedance tomography revealed that comparable high PEEP levels were needed to stabilize gas exchange area while hyperinflation of the lung was avoided [24]. Further the improvement in oxygenation is likely of clinical relevance, as indicated by previous work of Goligher and colleagues who demonstrated that a positive oxygenation response to increased PEEP is associated with a reduction of the probability of death in patients with severe ARDS [25]. Consistent with our findings, a recent retrospective analysis of a large COVID-19 ICU cohort from Northern Italy reported that ventilated patients require high PEEP levels for adequate oxygenation within the first 24 h after ICU admission [26].

Mechanistically, fluid accumulation may play an important role in the early disease stage in COVID-19 ARDS, as suggested by lung ultrasound, and opacities by CXR and CT scan [27]. Via functional loss of gas exchange area, increased diffusion distance, and ventilation-perfusion inhomogeneities, alveolar edema can contribute to severe hypoxemia in early COVID-19 ARDS, yet without necessarily increasing lung elastance as increased interalveolar strain may be counterbalanced by reduced surface tension [28]. The fact that high PEEP can re-aerate and thus functionally recruit fluid-filled alveoli in such a scenario [29] may in part explain the marked improvement in oxygenation in “L-type” lungs.

In contrast to the effects of mechanical ventilation *per se*, our finding that prone positioning markedly improved oxygenation did not appear to be related to lung recruitment as detectable by CXR. More homogeneous ventilation–perfusion matching and trans-pulmonary pressure distribution, regional changes in ventilation, as well as changes in chest wall mechanics may account for the oxygenation benefits of prone positioning in our patients [28,[30], [31], [32], [33]].

We analyzed CXR to assess lung recruitment. Compared to CT scans CXR have both shortcomings and advantages. CXR cannot dissect the exact underlying changes in the lungs as the CXR displays an anterior–posterior overlay image. Thus, further differentiation regarding radiographic phenotypes is not possible. Besides its shortcomings CXR was suitable to detect lung recruitment in our patients and may be considered of high value especially in the context of the current pandemic situation. (i) In daily routine CXR can be performed frequently; (ii) CXR demand less logistic and personal resources as compared to a CT scan. (iii) Further, intrahospital transportation to realize a CT scan for COVID-19 patients comprising the risk of contamination and infection of employees or other patients can be avoided by portable CXR machines. (iv) CXR are available comprehensively in most healthcare systems worldwide which is not the case for CT scans. This altogether underscores the value of our results for intensivists in nearly all health care systems especially in the current situation.

Our study has important limitations The number of investigated patients was relatively small. Importantly, however, due to the homogeneity of the studied cohort and the overall similar course of the early phase of COVID-19 related ARDS this small number was sufficient to address the key issue that high PEEP levels and the prone position in was effective in improving oxygenation in COVID-19 ARDS patients. As an initial observational study, focusing on the early phase of COVID 19 related ARDS, our study was not designed to assess outcome, and it is well known that improvements in oxygenation do not necessarily translate into decreased mortality. While we consider that our study provides important clues for mechanical ventilation in early COVID-19 ARDS, finally large multi-center randomized trials are necessary to determine the best ventilation strategies and their impact on outcome relevant parameters in this disease.

In summary, we demonstrate that patients with early COVID-19 ARDS can benefit in terms of oxygenation from mechanical ventilation with high PEEP as well as from prone positioning. Our findings provide evidence that may help guide intensivists in the treatment of early COVID-19 ARDS, and lend support to the pointed statement by Rice and Janz [34] that we should be cautious in withholding proven beneficial therapeutic strategies (e.g. PEEP and prone position) without evidence for patients with COVID-19 ARDS despite its remarkably unique nature.

## Data sharing statement

Anonymised patients data that underlie the results (tables, figures) reported in this study are available upon reasonable request to the corresponding author.

## Contributors

MM, AU, WMK, and HMR designed the design. MM, LBJ, AU, CG, FM, PP, AB, YL, FB, SW, and PK collected the data. MM, PP, FK, MD, FD, SWC, ASS, WMK, NS, and HMR were involved in data analysis. All authors were involved in the interpretation of the data and in the writing and critical review of the manuscript.

## Declaration of Competing Interest

MD is European Society of Radiology (ESR) Research Chair (2019–2022) and the opinions expressed in this publication are the author's own and do not represent the view of ESR. MD gives lectures for Canon, and Guerbet, and holds hands-on cardiac CT courses (www.ct-kurs.de). He is Editor of Cardiac CT (Springer Nature). He has institutional research agreements with Siemens, General Electric, Philips, Canon, and has a patent on fractal analysis of perfusion imaging (jointly with Florian Michallek, PCT/EP2016/071551).

All other authors declare no conflict of interest.

## Appendix Supplementary materials

Image, application 1
