# Supplementary material for: Evaluation of PEEP and prone positioning in early COVID-19 ARDS
Source: eClinicalMedicine. 2020 Oct 11;28:100579. doi: 10.1016/j.eclinm.2020.100579 (PMC7547915; doi:10.1016/j.eclinm.2020.100579)
Supplement: Supplementary file 1 [file mmc1.pdf]

Supplementary Appendix

This appendix has been provided by the authors to give readers additional information about their work.

Appendices

Figure S1: Chest x-rays prior and post intubation ....

Figure S2: Opacity score and chest x-rays before and after the first, second and third prone position ....

Table S1: Arterial blood gases, ventilator settings, and respiratory-system mechanics ....

Table S2: Overview of respiratory parameters and arterial blood gases for each step of the decremental PEEP trial for each of the n=7 patients ....

Table S3: Oxygenation, respiratory system compliance, and PEEP in prone vs. supine position............................

Supplementary Figure 1

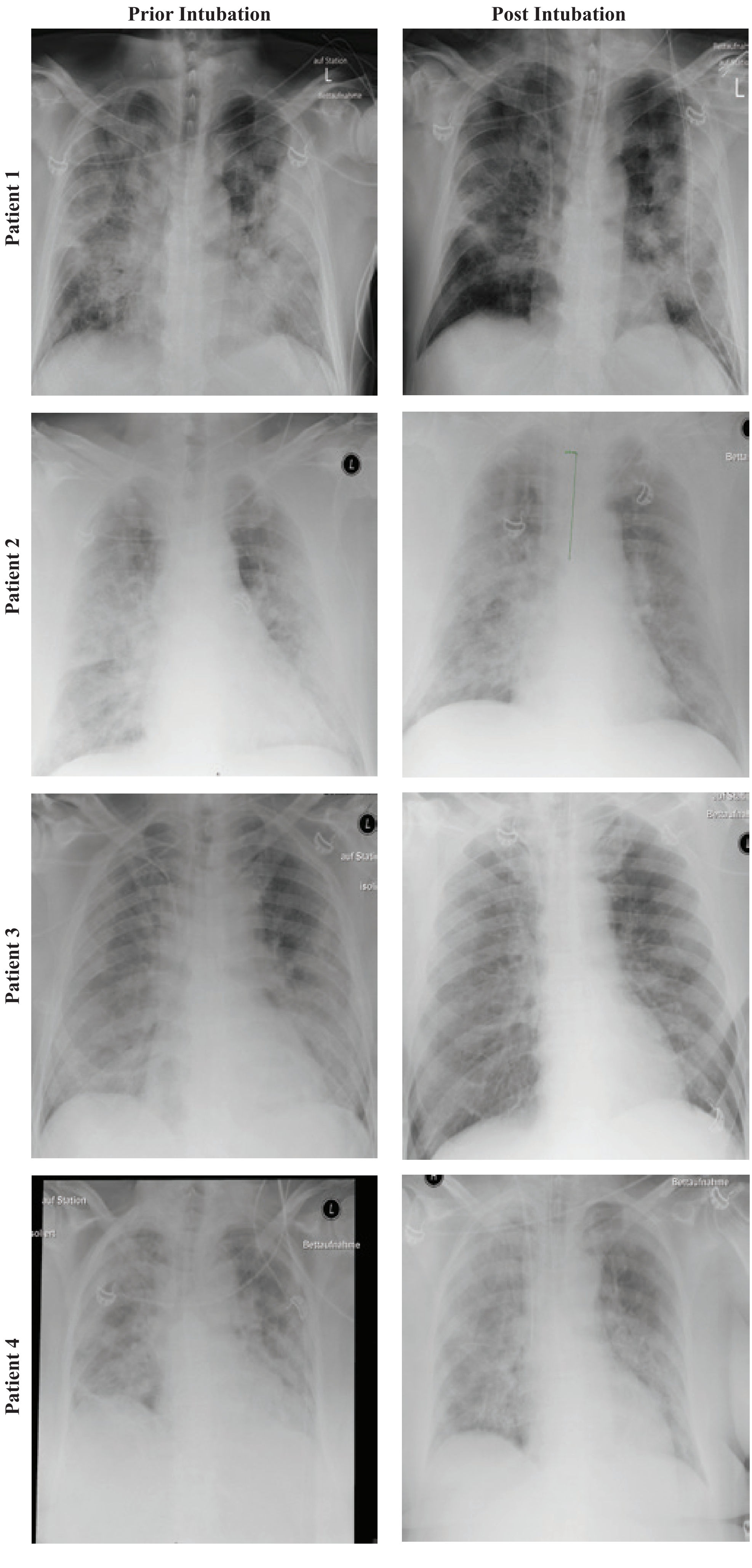

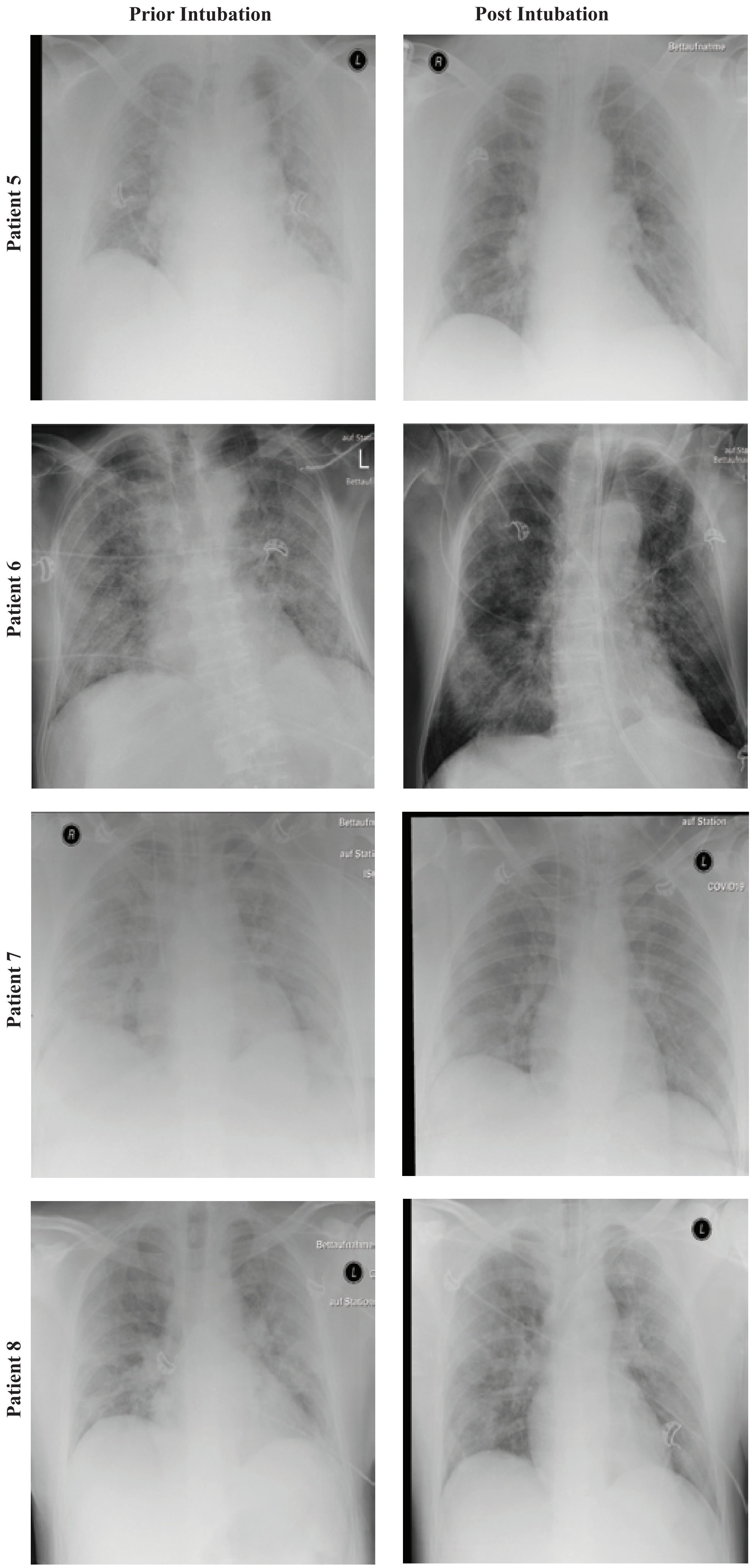

Prior Intubation
Post Intubation
Patient 5
Patient 6
Patient 7
Patient 8

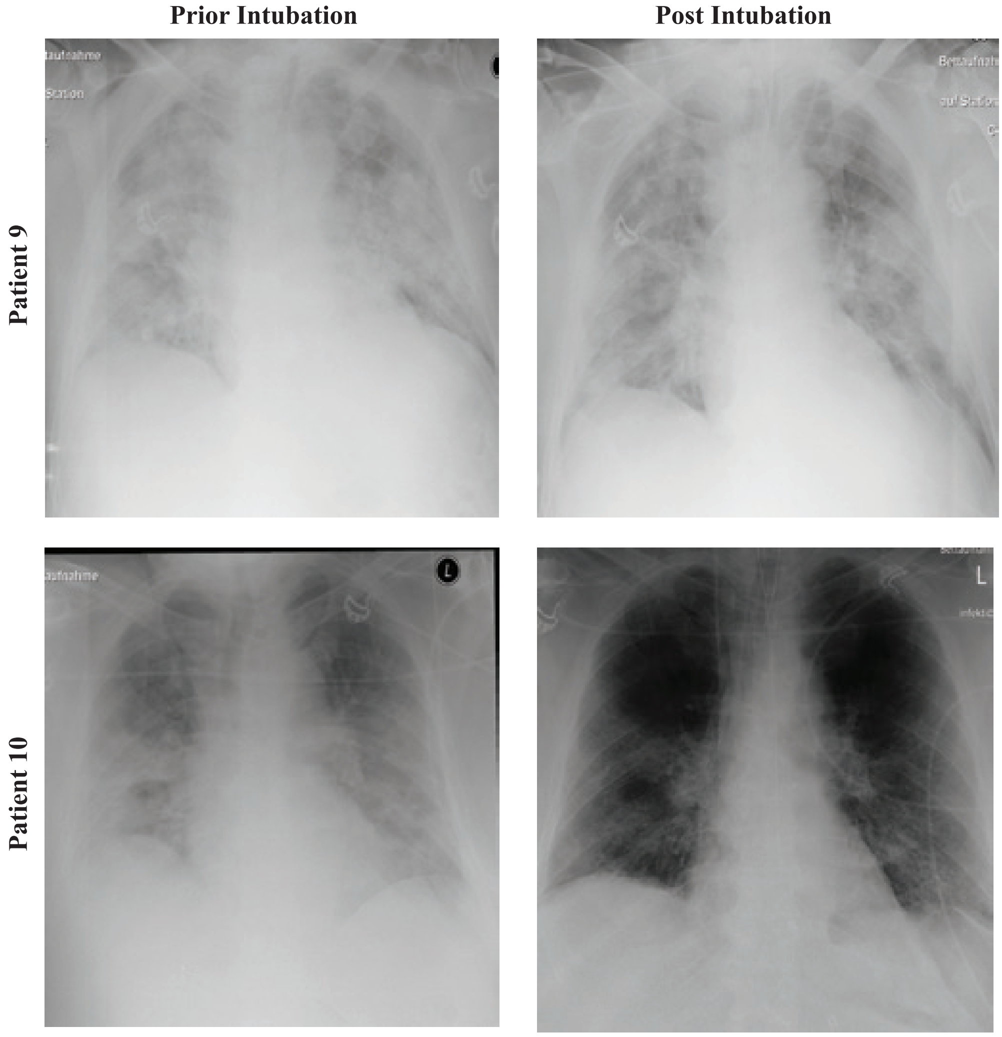

Supplementary Figure 1: Chest X-rays were taken prior intubation and between 3h to 16h post intubation.

Supplementary Figure 2

To observe whether pulmonary opacification changed after prone positioning, the opacity score was calculated and analyzed with a Wilcoxon signed-rank test. P values were not significant (A). Where available, chest x-rays are shown in supine position (SP) 2h to 24h prior the first prone positioning (PP), and after the first, second, and third PP (B). n.s. = not significant

A

	Prior PP	Post First PP	P value	Post 2nd PP	Post 3rd PP	P value
**Opacity Score**	23·7	22·5	n.s. (0·35)	22·0	22·3	n.s. (0·37)

B

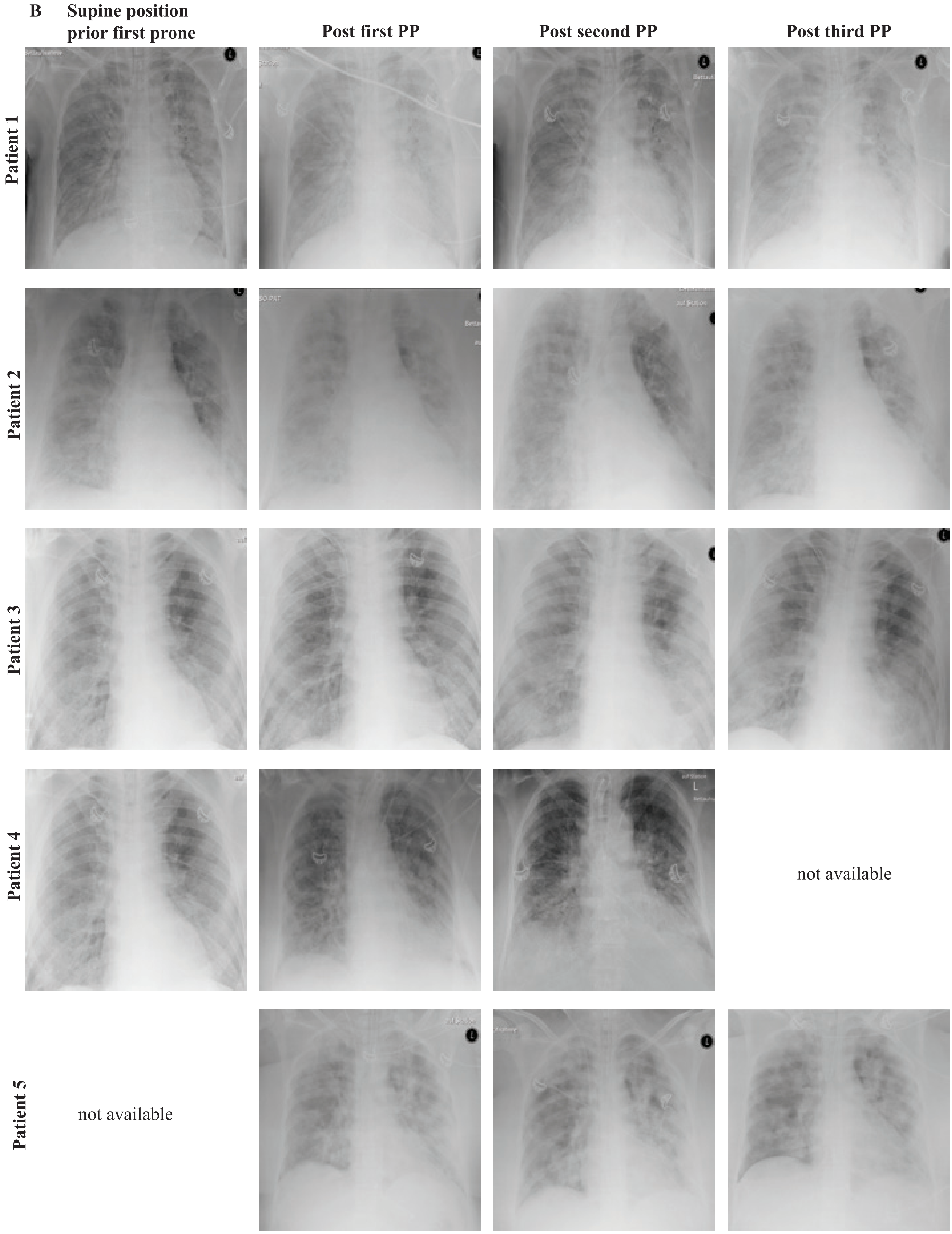

B

Supine position prior first prone | **Post first PP** | **Post second PP** | **Post third PP**

Patient 6 — not available

Patient 7

Patient 8

Patient 9

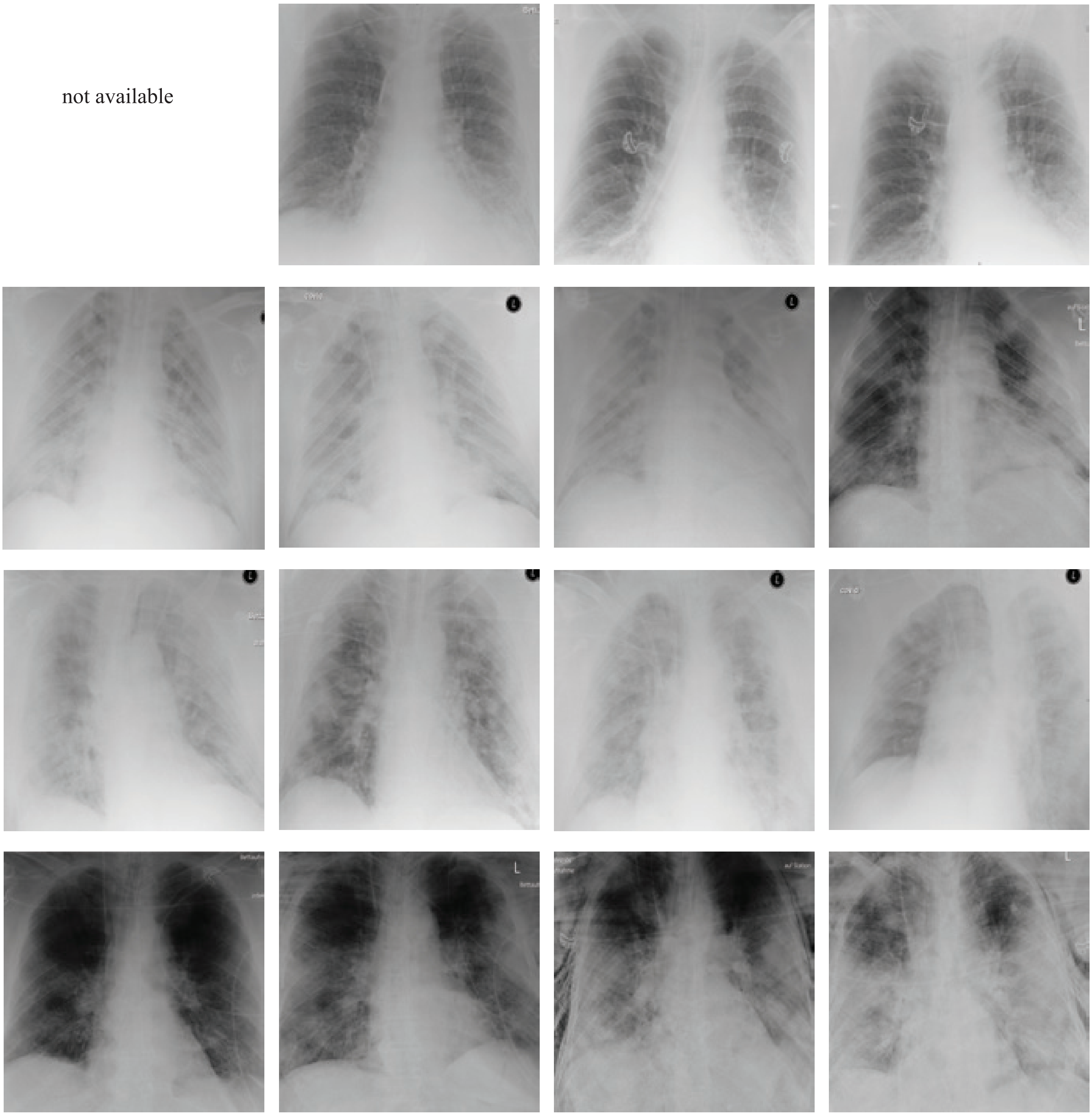

Table S1: Arterial blood gases, ventilator settings, and respiratory-system mechanics

	2 h Prior-Intubation Mean (± SD)	6 h Post-Intubation Mean (± SD)	P value of Mean (± SD)	2 h Prior-Intubation Median	6 h Post-Intubation Median	2 h Prior-Intubation Interquartile range	6 h Post-Intubation Interquartile range
**P_aO_2/F_iO_2 ratio (mmHg)**	84·3 ± 28·0	210·7 ± 86·6	0.0008	78·0	205·5	47·0	84·3
**F_iO_2**	0·8 ± 0·12	0·4 ± 0·11	<0.0001	0·8	0·41	0·16	0·22
**p_aCO_2 (mmHg)**	35·9 ± 7·0	43·1 ± 4·3	0.052	34·5	43·3	6·9	7·2
**Respiratory rate (min^{-1})**	31·0 ± 2·6	17 ± 2·3	<0.0001	32·0	16·0	3·8	4·5
**Peak Airway Pressure (mbar)**		30·1 ± 2·9			30·0		4·8
**PEEP (mbar)**		17·4 ± 3·6			19·0		5·5
**V_T (ml/kg)**		6·5 ± 0·5			6·5		0·55

P_aO_2/F_iO_2 ratio, respiratory rate, and ventilator settings were measured two hours prior and six hours post intubation from the ten patients of Subset 1. V_T, tidal volume. p-values as derived from two-sided t-test are shown.

Table S2: Overview of respiratory parameters and arterial blood gases for each step of the decremental PEEP trial for each of the n=7 patients.

**Patient 1**	**PEEP Trials in Supine Position and 12 to 24 h post Intubation**				
	**PEEP at start PEEP Trial**	**2nd PEEP**	**3rd PEEP**	**4th PEEP**	**5th PEEP**
PEEP (mbar)	24	**22**	20	18	
Fixed tidal volume (VT) (ml/kg)	420	**420**	420	420	
Respiratory rate (min^{-1})	13	**13**	13	13	
F_iO_2	0·5	**0·5**	0·5	0·5	
Respiratory system compliance (C_{RS}) (cmH_2O)	41	**59·6**	60	44·5	
Driving pressure (ΔP= end-inspiratory plateau pressure – PEEP) (mbar)	10	**8**	6	7	
Inspiratory trans-pulmonary pressure (TP_i= (end-inspiratory airway plateau pressure – end-inspiratory esophagus pressure) (mbar)	14	**10**	3·4	1	
End-expiratory trans-pulmonary pressure (TP_{exp}= PEEP - end-expiratory esophageal pressure) (mbar)	5·6	**3·1**	-1·7	-7·9	
Trans-pulmonary driving pressure ($\Delta TP=(TP_i-TP_{exp})$ (mbar)	8·4	**6·9**	5·1	8·9	
P_aCO_2 (mmHg)	60	**59**	59	53	
P_aO_2 (mmHg)	85	**88**	73	52	
**Patient 2**					
PEEP (mbar)	13	**11**	9		
Fixed tidal volume (VT) (ml/kg)	450	**450**	450		
Respiratory rate (min^{-1})	15	**15**	15		
F_iO_2	0·35	**0·35**	0·35		
Respiratory system compliance (C_{RS}) (cmH_2O)	41	**45**	45		
Driving pressure (ΔP= end-inspiratory plateau pressure – PEEP) (mbar)	11	**9**	9		
Inspiratory trans-pulmonary pressure (TPi= (end-inspiratory airway plateau pressure – end-inspiratory esophagus pressure) (mbar)	9·7	**8·2**	5·6		
End-expiratory trans-pulmonary pressure (TP_{exp}= PEEP - end-expiratory esophageal pressure) (mbar)	2·4	**0·3**	-1·5		
Trans-pulmonary driving pressure ($\Delta TP=(TP_i-TP_{exp})$ (mbar)	7·3	**7·9**	7·1		
P_aCO_2 (mmHg)	45	**44**	44		
P_aO_2 (mmHg)	86	**85**	85		
**Patient 3**					
PEEP (mbar)	24	22	**20**	18	
Fixed tidal volume (VT) (ml/kg)	550	550	**550**	550	
Respiratory rate (min^{-1})	13	13	**13**	13	
F_iO_2	0·3	0·3	**0·3**	0·3	
Respiratory system compliance (C_{RS}) (cmH_2O)	60	70	**79**	73	

Driving pressure (ΔP= end-inspiratory plateau pressure – PEEP) (mbar)	7	7	**6**	7	
Inspiratory trans-pulmonary pressure (TP_i= (end-inspiratory airway plateau pressure – end-inspiratory esophagus pressure) (mbar)	9·7	7·6	**5·3**	4·7	
End-expiratory trans-pulmonary pressure (TP_{exp}= PEEP - end-expiratory esophageal pressure) (mbar)	4	2·3	**0·8**	-0·6	
Trans-pulmonary driving pressure ($\Delta TP=(TP_i-TP_{exp})$	5·7	5·3	**4·5**	5·3	
P_aCO_2 (mmHg)	54	52	**53**	50	
P_aO_2 (mmHg)	79	74	**71**	65	
**Patient 4**					
PEEP (mbar)	**18**	16	14	12	10
Fixed tidal volume (VT) (ml/kg)	**420**	420	420	420	420
Respiratory rate (min^{-1})	**15**	15	15	15	15
F_iO_2	**0·35**	0·35	0·35	0·35	0·35
Respiratory system compliance (C_{RS}) (cmH_2O)	**64·8**	66·4	51·3	50·4	44·7
Driving pressure (ΔP= end-inspiratory plateau pressure – PEEP) (mbar)	**11**	9·3	10	9·5	9·5
Inspiratory trans-pulmonary pressure (TP_i= (end-inspiratory airway plateau pressure – end-inspiratory esophagus pressure) (mbar)	**15**	13	12	11	9
End-expiratory trans-pulmonary pressure (TP_{exp}= PEEP - end-expiratory esophageal pressure) (mbar)	**10**	9·2	8·2	7	5·5
Trans-pulmonary driving pressure ($\Delta TP=(TP_i-TP_{exp})$ (mbar)	**7·1**	6	5·1	5·6	6·4
P_aCO_2 (mmHg)	**47·3**	44·8	46·2	43·5	46·1
P_aO_2 (mmHg)	**65·3**	58·1	59·8	58·4	
**Patient 5**					
PEEP (mbar)	20	18	**16**	14	
Fixed tidal volume (VT) (ml/kg)	320	320	**320**	320	
Respiratory rate (min^{-1})	22	22	**22**	22	
F_iO_2	0·5	0·5	**0·5**	0·5	
Respiratory system compliance (C_{RS}) (cmH_2O)	25·4	31	**30**	38	
Driving pressure (ΔP= end-inspiratory plateau pressure – PEEP) (mbar)	19	17	**17·2**	16	
Inspiratory trans-pulmonary pressure (TP_i= (end-inspiratory airway plateau pressure – end-inspiratory esophagus pressure) (mbar)	18	15	**13·8**	12·9	
End-expiratory trans-pulmonary pressure (TP_{exp}= PEEP - end-expiratory esophageal pressure) (mbar)	34·5	11	**10·8**	11	
Trans-pulmonary driving pressure ($\Delta TP=(TP_i-TP_{exp})$ (mbar)	13·5	11	**11·8**	11	
P_aCO_2 (mmHg)	76·4	74·9	**73·4**	69·8	
P_aO_2 (mmHg)	65·2	67·7	**81·7**	67·8	
**Patient 6**					
PEEP (mbar)	18	**16**	14		
Fixed tidal volume (VT) (ml/kg)	420	**420**	420		
Respiratory rate (min^{-1})	16	**16**	16		

F_iO_2	0·6	**0·6**	0·6		
Respiratory system compliance (C_{RS}) (cmH_2O)	35·5	**41·9**	43·3		
Driving pressure (ΔP= end-inspiratory plateau pressure – PEEP) (mbar)	12	**11**	11		
Inspiratory trans-pulmonary pressure (TP_i= (end-inspiratory airway plateau pressure – end-inspiratory esophagus pressure) (mbar)	15	**11**	10		
End-expiratory trans-pulmonary pressure (TP_{exp}= PEEP - end-expiratory esophageal pressure) (mbar)	4·6	**2·6**	1·3		
Trans-pulmonary driving pressure ($\Delta TP=(TP_i-TP_{exp})$ (mbar)	11·4	**8·4**	8·7		
P_aCO_2 (mmHg)	47·5	**45**	44·5		
P_aO_2 (mmHg)	79·1	**79·6**	71·9		
**Patient 7**					
PEEP (mbar)	24	**22**	20	18	
Fixed tidal volume (VT) (ml/kg)	500	**500**	500	500	
Respiratory rate (min^{-1})	16	**16**	16	16	
F_iO_2	0·35	**0·35**	0·35	0·35	
Respiratory system compliance (C_{RS}) (cmH_2O)	52·5	**60·1**	69	73	
Driving pressure (ΔP= end-inspiratory plateau pressure – PEEP) (mbar)	9	**9**	8	8	
Inspiratory trans-pulmonary pressure (TP_i= (end-inspiratory airway plateau pressure – end-inspiratory esophagus pressure) (mbar)	15	**14**	12	11	
End-expiratory trans-pulmonary pressure (TP_{exp}= PEEP - end-expiratory esophageal pressure) (mbar)	8·7	**7·9**	6·7	6	
Trans-pulmonary driving pressure ($\Delta TP=(TP_i-TP_{exp})$ (mbar)	6·3	**6·1**	5·3	5	
P_aCO_2 (mmHg)	50	**48·4**	49·2	49	
P_aO_2 (mmHg)	84·3	**88·7**	88	86	

PEEP trials were performed between 2h and 60h after initiation of mechanical ventilation. The optimal PEEP and the corresponding values are highlighted in bold.

Table S3: Oxygenation, respiratory system compliance, and PEEP in prone vs. supine position

	First SP Mean ± SD	First PP Mean ± SD	P value Mean ± SD	First SP Median	First PP Median	First SP IQR	First PP IQR
**P_aO_2/F_iO_2 ratio (mmHg)**	118·4 ± 41·9	181·8 ± 63·2	0·03	114·0	162·0	55·0	77·0
**Oxygenation index (OI)**	23·8 ± 8·2	14·7 ± 5·7	0·04	23·0	14·0	12·0	10·0
**PEEP**	20·1 ± 2·4	18·6 ± 4·3	n.s. (0·15)	20·0	20·0	4·0	5·5
	**First three SP Mean ± SD**	**First three PP Mean ± SD**	**P value Mean ± SD**	**First three SP Median**	**First three PP Median**	**First three SP IQR**	**First three PP IQR**
**P_aO_2/F_iO_2 ratio (mmHg)**	145·7 ± 48·8	192·2 ± 32·7	0·03	128·0	193·0	11·0	18·0
**Oxygenation index (OI) (mbar/mmHg)**	20·8 ± 7·2	13·2 ± 3·4	0·01	20·0	13·0	5·0	3·0
**PEEP (mbar)**	19·9 ± 2·4	17·8 ± 3·4	0·01	20·0	18·0	2·0	2·0
	**All SP Mean ± SD**	**All PP Mean ± SD**	**P value Mean ± SD**	**All SP Median**	**All PP Median**	**All SP IQR**	**All PP IQR**
**P_aO_2/F_iO_2 ratio (mmHg)**	136·9 ± 23·5	191·6 ± 36·1	0·002	127·0	191·0	31·0	56·0
**Oxygenation index (OI) (mbar/mmHg)**	19·7 ± 4·2	13·6 ± 2·8	0·004	20·0	14·0	9·0	4·0
**PEEP (mbar)**	19·2 ± 2·0	17·3 ± 3·3	0·02	20·0	18·0	3·0	4·0

Data were recorded after 4h in supine positioning (SP) and 12h in prone positioning. Mean values ± standard deviation are given for the first prone positioning (first SP vs. first PP), after the first three PP maneuvers (first three SP vs. first three PP), and for all prone positions (all SP vs. all PP). p-values as derived from two-sided t-test are shown. Abbreviation: n.s. = not significant. IQR: Interquartile range.
